# Editorial: The Fifth Konstantin Ivanov Intercontinental Magnetic Resonance Conference on Methods and Applications ICONS-5

**DOI:** 10.1007/s00723-022-01501-y

**Published:** 2022-09-23

**Authors:** G. Buntkowsky, D. Abergel, P. K. Madhu

**Affiliations:** 1 Institute of Inorganic and Physical Chemistry, Technical University of Darmstadt, 64287 Darmstadt, Germany; 2 Laboratoire des Biomolécules, Département de Chimie, École Normale Supérieure, PSL, University, CNRS, Sorbonne Université, 75005 Paris, France; 3 TIFR Centre for Interdisciplinary Sciences, Tata Institute of Fundamental Research Hyderabad, 36/P Gopanpally Village, Ranga Reddy District, Hyderabad, 500107 India

Give Peace a Chance

John Lennon 1969

ICONS-5, organized during from August 31 to December 02, 2022, was the fifth edition of the on-line magnetic resonance conference series called Konstantin Ivanov InterCONtinental Magnetic Resonance Seminar, named after our untimely deceased colleague and friend. The ICONS conferences are an off-shoot of the weekly Intercontinental NMR Seminar Series that started on April 8, 2020. This seminar series has enabled the communication and dissemination of research ideas among the magnetic research community in the times of the COVID-19 pandemic and will continue to do so beyond. In the framework of the ICONS series, until now, more than 130 scientists from five different continents have presented their recent results. While the weekly seminar series gives both early-stage and experienced researchers an opportunity to give seminar talks and interact with colleagues from all over the world, the ICONS conference is a platform for experienced researchers. The ICONS-5 conference attracted registrations from nearly 200 people from 30 countries (in the spirit of the meeting, covering 6 continents, Europe, North America, South America, Africa, Australia, and Asia) and spanned 17 time zones from Japan over Europe to the US West Coast. The meeting talks were broadcast across the Zoom and YouTube platforms. The average combined attendance was around 120.

The ICONS seminar series is open to all areas of magnetic resonance and covers the full range of Magnetic Resonance, i.e., EPR, NMR, MRI, and their various hybrids. While the summer ICONS conferences (see reports in APMR [1, 2] for details) are equally broad in scope as the seminars, the spring conferences are focused on a narrower subject, such as techniques, where the interaction of electron and nuclear spins play a pivotal role (ICONS-2) [3] for details or the various flavors of hyperpolarization and NMR signal enhancement (ICONS-4) [4]. The main goal of ICONS-5 was to present an overview about the current state-of-the-art of various fields of Magnetic Resonance, ranging from pulse-sequence development over hyperpolarization like DNP and PHIP and single-spin ESR to the study of dynamic processes like relaxation, exchange processes, phase separation and diffusion with the idea to further support and stimulate interactions among groups employing these techniques in EPR, NMR and MRI. To achieve this goal, twelve speakers were selected among the leading experts in these fields and invited to report at the conference.

Malcolm Levitt, UK, reported on the utilization of symmetry-based strategies designed for the optimization of pulse-sequences in solid-state NMR towards applications in solution NMR spectroscopy. For this, he first gave a clear introduction into symmetries of pulse-sequences, such as time reversal and their effects on the average Hamiltonian created by the pulse-sequence, employing RN_n_^v^ sequences as a prominent example. Then he introduced a new symmetry-based sequence for the conversion of singlet to triplet spin order and discussed the robustness of the new sequence.

Björn Corzilius, Germany, gave first an overview about the techniques and applications of Dynamic Nuclear Polarization (DNP) and its pros and cons compared to other hyperpolarization techniques. Then he reported on recent developments on the application of DNP towards studies of RNA ribozymes and discussed the application potential of DNP for the simplification of crowded spectra of biomolecules. At the end, he gave an overview of ^15^N-DNP NMR employing Gd^3+^ radicals.

Paul Schanda, Austria, gave a fascinating overview of recent developments in the investigation of protein dynamics in the solid state employing a combination of solid-state NMR and molecular dynamics simulations. At the start of his presentation, he explained why relaxation is intrinsically multi-exponential. Then he discussed the effects of deuteration on the relaxation times and gave a Redfield analysis of R_1ρ_. At the end, he reported a combination of REDOR and VT-NMR studies of side-chain dynamics of proteins.

Thomas Theis, USA, first gave an introduction into Parahydrogen-based hyperpolarization techniques in Chemistry, Biochemistry, and medical imaging. He then discussed the advantages of reversible PHIP (SABRE), in particular in bio-related questions, owing to the large number of available metabolites of interest that are or are likely to become SABRE active, and showed a number of recent results from his group. Next, he gave a fascinating overview about the construction and physical principles of a RASER NMR setup in his group and demonstrated the astounding coherence length and spectral resolution of this system and the resulting line resolution.

Jamie Walls, USA, discussed the application potential of diffusion NMR for the investigation of NMR diffusion experiments for the analysis of the pharmaco-kinetics of emulsion-based formulations of drugs and its application to isoflurane, a widely employed common anesthetic. He discussed the kinetics employing Bloch-McConnell model.

Patrice Bertet, France, gave a fascinating introduction into recent results in the detection of small numbers of electron spins employing micro-waves in a miniaturized resonator. He explained how the relaxation properties of the spins are optimized and discussed the SMPD device for the detection of the signals. Then he showed the tremendous improvements obtained in the last twelve month where the detection limit was increased from 1000 to one spin. Finally, he showed a number of basic experiments, such as Rabi oscillations on these systems.

Ashok Sekhar, India, discussed the application of NMR for the investigation of order/disorder phase transitions in intrinsically disordered proteins, employing the cytidine repressor, which binds its cognate operator DNA through the N-terminal DNA-binding domain (CytR^N^). By CEST (Chemical Exchange Saturation Transfer) an excited state of the protein was detected, whose structure was studied via ^1^H-^15^N RDCs (residual dipolar couplings).

Bernhard Blümich, Germany, gave an exciting analysis of the jump dynamics in three-site exchange by analyzing T_2_–T_2_ exchange NMR and its connections to the principle of detailed balance. He showed that the inclusion of wall interactions with the exchanging particles can lead to a preferential circular motion of a small fraction of the particles, which he interprets as a manifestation of higher diffusion eigenmodes.

Nikolai Skrynnikov, Russia, reported on new developments in the area of diffusion NMR for protein systems. First, he gave a short introduction into diffusion NMR and the chemistry of fiber forming Sup35NM fragments, which are related to amyloido-genesis. He showed that it is feasible to separate spectral signals from fibrils and other species that may be present in the sample by means of their dynamics. Then he introduced a novel, WEB-based software (DDfit) for the processing of diffusion NMR spectra.

Jean-Pierre Korb, France, reported on recent studies of water dynamics in protein hulls, employing a multi-scale approach. From the combination of the experimental dispersion of the NMR relaxation data and theoretical modeling he investigated the surface water diffusion in hen egg-white lysozyme (LZM) and bovine serum albumin (BSA) solutions and the influence of the addition of different salts on these dynamics. He found an excellent agreement between experiments and modeling over 4 decades in Larmor frequency (10 kHz to 110 MHz).

Eva Meirovitch, Israel, gave an intense theoretical introduction into the combination of the slowly relaxing local structure (SRLS) approach for the analysis of NMR relaxation from proteins and molecular dynamics (MD) simulations and the application of these techniques towards the study of NH-bonds.

Thomas Barbara, USA, gave a fascinating historical overview about the development of NMR relaxation theories. In this overview, he showed that already Felix Bloch had developed a full density matrix approach for the description of relaxation, which later fell into oblivion in favor of Redfield's simpler semiclassical approach and how this old approach by Bloch is related to the recent rediscovery of the Lindblad master equation.

Organization and future developments: The conference was organized by Daniel Abergel (ENS Paris, France), Gerd Buntkowsky (TU, Darmstadt, Germany), and P. K. Madhu (TIFR Hyderabad, India). Suman Saurav, TIFR Hyderabad, provided technical assistance. The conference and seminar series were sponsored by Alexander von Humboldt Foundation, Wiley, Springer, HyperSpin, and Adani. Following the scheme of a general MR conference in summer alternating with a specialized conference on cutting-edge topics in winter, there are already plans for a specialized ICONS-6 in spring of 2023. For updates and the schedule of upcoming talks, see the home page of the meeting ICONS-Seminar.
